# Clinical Characteristics of Nursing- and Healthcare-Associated Tuberculosis

**DOI:** 10.3390/diseases6040101

**Published:** 2018-11-11

**Authors:** Toshitaka Suzuki, Tatsuo Kato, Ryoko Ohnishi, Shigeo Yasuda, Kimiyasu Sano, Yohei Shirakami, Masahito Shimizu, Nobuo Murakami

**Affiliations:** 1 Department of Respiratory Medicine, National Hospital Organization Nagara Medical Center, 1300-7 Nagara, Gifu 502-8558, Japan; toshi-1976-su@nagara-lan.hosp.go.jp (T.S.); katot@nagara-lan.hosp.go.jp (T.K.); ryoko@nagara-lan.hosp.go.jp (R.O.); yasudas@nagara-lan.hosp.go.jp (S.Y.); kimisano@nagara-lan.hosp.go.jp (K.S.); 2 Department of Informative Clinical Medicine, Gifu University Graduate School of Medicine, 1-1 Yanagido, Gifu 501-1194, Japan; 3 Department of Gastroenterology/Internal Medicine, Gifu University Graduate School of Medicine, 1-1 Yanagido, Gifu 501-1194, Japan; shimim-gif@umin.ac.jp; 4 Biological Support Center, Gifu University Hospital, 1-1 Yanagido, Gifu 501-1194, Japan; muranobi@gifu-u.ac.jp

**Keywords:** elderly tuberculosis patients, community-acquired tuberculosis, NHCAP

## Abstract

Tuberculosis remains a serious health problem worldwide. Patients with tuberculosis who also require nursing care due to aging and underlying diseases are considered to have a high mortality rate; however, there are few studies describing detailed examinations of such disease conditions. Objective: The present study was conducted to investigate differences in clinical features of elderly tuberculosis patients according to the levels of nursing and healthcare required. Design: The study participants included 146 elderly (≥65 years) patients diagnosed with active tuberculosis among patients hospitalized with tuberculosis at a single center. The patients were classified into two groups: a nursing- and healthcare-associated tuberculosis group (n = 71) and a community-acquired tuberculosis group (n = 75). Results: The nursing- and healthcare-associated tuberculosis patients were older and had a higher frequency of comorbidities compared with the community-acquired tuberculosis group. Patients in the nursing- and healthcare-associated tuberculosis group had markedly lower levels of serum albumin and hemoglobin, and higher levels of C-reactive protein. The rate of in-hospital death was significantly higher in the nursing- and healthcare-associated tuberculosis group. This was attributed to malnutrition and comorbid conditions rather than the severity of tuberculosis. Conclusion: The prognosis was poor in elderly tuberculosis patients receiving nursing and healthcare.

## 1. Introduction

Tuberculosis (TB) remains one of the top ten causes of death worldwide. The estimated number of deaths from TB was 1.4 million in 2015, although the total number of deaths from TB is now decreasing [1]. The incidence of TB also continues to decline slightly; however, TB in the elderly, a population who are considered high-risk, is increasing due to longevity. This is especially true in industrialized countries [2]. These trends are also seen in Japan, where the annual infection rate of TB is decreasing, however, a large number of elderly TB patients have been newly diagnosed and these elderly patients comprise 57.4% of total TB patients [3,4]. It is thought that as the population continues to age, measures to control TB in the elderly are needed. In particular, when considering the present social and medical situation, the increasing population of elderly TB patients who require healthcare or nursing care appears to be a serious medical care problem.

In 2011, the Japanese Respiratory Society (JRS) proposed a new concept, nursing- and healthcare-associated pneumonia (NHCAP) [5]; this considers the Japanese healthcare environment and insurance system while modifying the healthcare-associated pneumonia guidelines jointly published by the American Thoracic Society and Infectious Diseases Society of America [6]. In elderly pneumonia patients, community-acquired pneumonia and NHCAP are caused by different pathogenic bacteria and have different prognoses. NHCAP is a drug-resistant pneumonia and has poor outcomes in elderly patients [7]. Several clinical factors, including advanced age (≥80 years), are reported as adverse prognosis factors for NHCAP patients [7].

Similar to the pneumonia cases, it is strongly expected that elderly TB patients who require nursing and healthcare may also have a high mortality rate. However, detailed research examining the characteristics of elderly TB patients, such as clinical features and prognosis, according to required levels of nursing and healthcare have not yet been conducted. In the present study, we defined TB patients requiring healthcare and nursing care as having “nursing- and healthcare-associated TB (NHTB)” and investigated the clinical characteristics of elderly patients with NHTB.

## 2. Materials and Methods

### 2.1. Patients

The participants included 146 patients aged ≥65 years and diagnosed with active TB among a pool of 200 TB patients hospitalized at the National Hospital Organization Nagara Medical Center between 1 January, 2010, and 31 December, 2011. Ninety-two (46%) patients hospitalized in the TB ward at our medical facility were ≥80 years old.

### 2.2. Study Design

A retrospective study was conducted to investigate clinical features of both the NHTB and community-acquired TB (CTB) groups. The definitions of NHTB and CTB are in the results section.

The research followed was in accordance with the Declaration of Helsinki. All study participants or, when participants were already dead, their family members provided verbal informed consent. This consent procedure was considered sufficient because the present study followed an observational research design that did not need new human biological specimens, instead relying only on pre-existing clinical records and data. This study protocol was determined by following the Ethical Guidelines for Epidemiological Research (Ministry of Education, Culture, Sports, Science and Technology and Ministry of Health, Labour and Welfare in Japan). The study design, including this consent procedure, was approved by the institutional ethics committee of the National Hospital Organization Nagara Medical Center.

The following parameters in both groups were investigated: age and gender of the patient; response to a sputum smear test; levels of serum albumin, hemoglobin, and C-reactive protein (CRP); white blood cell (WBC), neutrophil, and lymphocyte counts at time of hospitalization; comorbid conditions; period of hospitalization; and outcome (survival or death).

### 2.3. Statistical Analysis

Statistical analysis was performed with StatMate III (ATMS Co., Ltd., Tokyo, Japan) using Chi-square or Student’s *t*-tests. *p* < 0.05 was considered significant.

## 3. Results

### 3.1. Definition and Classification of NHTB Patients

The definition of NHTB was established in reference to the JRS NHCAP clinical practice guidelines [5] The parameters were as follows: (1) TB diagnosed in a resident of an extended care facility or nursing home, (2) TB diagnosed in a person discharged from a hospital within the preceding 90 days, (3) TB diagnosed in an elderly or disable person receiving nursing care, and (4) TB diagnosed in a person receiving regular outpatient endovascular treatment by dialysis, antibiotics, anticancer agents, or immunosuppressants. CTB is defined as TB diagnosed in a person who maintains a life outside of the hospital. Patients in this study were grouped according to the definitions.

The definition of NHTB is summarized and listed in Table 1. Items 1 and 3 are identical to those listed in the NHCAP clinical practice guidelines. Items 2 and 4, pertaining to the hospitalization history of illnesses other than TB, and treatment with biological agents, respectively, were also included. Patients who met one of these criteria were included in the NHTB group, while the remaining patients were included in the CTB group. As shown in Table 1, all 71 patients in the NHTB group were classified into four categories using the NHTB definition: 15 patients (21.1%) in Category 1, 11 patients (15.5%) in Category 2, 42 patients (59.2%) in Category 3, and 3 patients (4.2%; 1 patient was treated with biological agents and 2 with anticancer agents) in Category 4.

### 3.2. Patient Characteristics and Laboratory Data

Patient characteristics and laboratory data at the time of hospitalization are listed in Table 2. The NHTB and CTB groups included 71 (35 men and 36 women) and 75 (59 men and 16 women) patients, respectively. The age of patients in the NHTB group was significantly higher than that of those in the CTB group (*p* < 0.01). There was no significant difference in the number of smear-positive patients among groups. The NHTB group had significantly lower levels of albumin and hemoglobin and higher levels of C-reactive protein (CRP) when compared with the CTB group (*p* < 0.01 in each comparison). No significant differences were observed in white blood cell (WBC), neutrophil, or lymphocyte counts between these groups. None of the patients were human immunodeficiency virus positive or suffering from multidrug-resistant TB.

### 3.3. Comorbid Conditions in NHTB and CTB Groups

As shown in Table 3, the NHTB group showed a remarkably higher frequency of comorbidities (diabetes, cerebrovascular disease, dementia, malignancy, and gastrectomy) than the CTB group (77.5% vs. 53.3%, *p* < 0.01). Among these underlying conditions, there was a significant difference between the two groups with respect to dementia (*p* < 0.01).

### 3.4. Prognosis of NHTB and CTB Groups

Patients in the NHTB group were hospitalized for 77.6 ± 42.5 days, which was significantly shorter than for those in the CTB group (94.0 ± 42.6 days, *P* = 0.02) (Table 4). The short period of hospitalization in the NHTB group might be explained by the high mortality rate in this group, as discussed in the following section. The rate of treatment discontinuation was 35.2% and 22.7% in the NHTB and CTB groups, respectively, but there was no significant difference. Analysis of disease prognosis revealed that the rate of in-hospital death was 39.4% in the NHTB group, which was significantly higher than the corresponding rate of 4.0% in the CTB group (*p* < 0.05).

## 4. Discussion

In recent years, the number of immune-compromised patients, i.e., hosts, in Japan has been steadily increasing due to the population continuing to age and healthcare practices becoming more sophisticated. These patients are at high-risk of acquiring infectious diseases, especially respiratory infections. For cases of pneumonia the JRS created the NHCAP guidelines, which apply to elderly pneumonia patients chiefly from long-term care facilities and nursing homes [5]. The NHCAP patients, who are closely related to healthcare institutions, have a poor prognosis and, therefore, require appropriate general care and treatment [8]. In addition, several studies have suggested that the poor prognosis of these patients is significantly associated with specific factors related to the host, such as their general health [9,10,11,12].

It has been suggested that the percentage of elderly TB patients showing a high mortality rate is increasing [2,4,13]. Therefore, medical treatment for elderly TB patients, especially those who require healthcare and nursing care, is becoming an important issue. In the present study, we investigated differences in clinical features stemming from the levels of healthcare and nursing care required by elderly TB patients aged ≥65 years at our medical facility; we then proposed a new concept, NHTB, which is based on the NHCAP guidelines, for elderly patients with pulmonary TB.

The results of the present study showed that 48.6% of elderly patients diagnosed with active TB at our medical facility could be included in the NHTB group (Table 2), and approximately 60% of patients in the NHTB group required nursing care due to physical disability. These were classified as Category 3 NHTB patients (Table 1). The prognosis for patients in the NHTB group was markedly poorer than for patients in the CTB group (Table 4). This poor prognosis appears to be associated with poor general health, including nutritional status, rather than the severity of the TB because the NHTB group had a higher frequency of comorbidities and lower levels of albumin and hemoglobin (Table 2 and Table 3). It has been suggested that most of the elderly pulmonary TB patients have comorbid disorders and show deterioration of nutritional status represented by low serum albumin levels and decreased dietary intake [14,15,16,17]. In addition, previous reports have indicated that poor nutritional status, reflected by low serum albumin and anemia, can be clinical predictors of early mortality from TB [18,19]. The results of our present study together with those of recent reports [16,18,19,20,21] strongly suggest that comorbid pathological conditions and malnutrition significantly worsen the prognosis of elderly patients with pulmonary TB, especially when they require healthcare and nursing care.

In the present study, the rate of in-hospital death was significantly higher in the NHTB group. Patients in the NHTB group had a markedly shorter period of hospitalization (Table 4); however, upon exclusion of fatal cases, significant differences between the two groups were no longer observed (94.3 ± 38.2 days for the NHTB group and 95.2 ± 42.0 days for the CTB group, *P* = 0.91). This might be attributable to the fact that approximately 40% of the patients in the NHTB group died within 30 days of hospitalization. Although patient age and mortality rate was higher in the NHTB group, the rate of in-hospital death in the CTB group was extremely low (4.0%, Table 4), suggesting that aging may have almost no influence on prognosis. Therefore, the present study on TB among the elderly might indicate that poor general health, especially malnutrition and comorbidities, has a greater effect on prognosis than the severity of the TB itself. These results are similar to those of the previous report comparing NHCAP with community-acquired pneumonia, in which patient backgrounds were deeply related to excess mortality in NHCAP patients [7]. Considering this aspect, the concept of NHTB is thought to be useful.

The major limitation of our present investigation is that it is a retrospective study with a relatively small number of participants at a single center. In addition, treatment bias caused by the general health of the patients and the intention of their family members cannot be completely avoided. Moreover, though this study was not focused on drug-resistant TB, drug resistance is often associated with the disease condition of NHCAP.

## 5. Conclusions

In conclusion, in the present study we proposed a new concept of NHTB for elderly TB patients based on the JRS NHCAP clinical practice guidelines [5]. In elderly TB patients, especially those included in the NHTB group, poor general health and the existence of comorbidities appeared to have a greater impact on prognosis than the severity of TB. Therefore, the concept of NHTB, which reflects the social situation and healthcare environment, may be feasible in considering medical care and treatment for TB.

## Figures and Tables

**Table 1:** Definition and characterization of patients with NHTB.

Category	Characterization	No. of Patients (%) (total n = 71)
(1)	Admission to a long-term care facility or nursing home (including a psychiatric ward).	15 (21.1)
(2)	Hospitalization for ≤90 days for an illness other than tuberculosis.	11 (15.5)
(3)	Elderly or physically disabled persons PS 3 requiring nursing care. PS 3 refers to patients having the ability to care for themselves only to a limited degree and who spend ≥50% of their day in bed or in a wheelchair.	42 (59.2)
(4)	Undergoing endovascular treatment, including dialysis, antibiotic therapy, chemotherapy, immunosuppressive therapy or continuous treatment using biological agents on an outpatient basis.	3 (4.2)

NHTB—nursing- and healthcare-associated tuberculosis; PS—performance status.

**Table 2:** Baseline characteristics and laboratory data of patients with NHTB and CTB.

Items	NHTB (n = 71)	CTB (n = 75)	*P*-Value
Age (range)	84.1 (67–101)	80.4 (66–95)	<0.01
Gender (male/female)	35/36	59/16	<0.01
Smear (positive/negative)	63/8	66/9	0.89
Albumin (g/dL)	2.53 ± 0.66 ^a^	3.33 ± 0.73	<0.01
Hemoglobin (g/dL)	10.7 ± 1.8	12.2 ± 1.9	<0.01
CRP (mg/dL)	5.90 ± 4.85	3.59 ± 4.11	<0.01
WBC count (/μL)	7880 ± 5361	7068 ± 3460	0.29
Neutrophil (/μL)	6090 ± 2873	5528 ± 3410	0.29
Lymphocyte (/μL)	1284 ± 4378	971 ± 538	0.55

^a^ Mean ± standard deviation. NHTB—nursing- and healthcare-associated tuberculosis; CTB—community-acquired tuberculosis; CRP—C-reactive protein; WBC—white blood cell.

**Table 3:** Comorbid conditions in patients with NHTB and CTB.

Items	NHTB (n = 71)	CTB (n = 75)	*P*-Value
Overall (%)	55 (77.5)	40 (53.3)	<0.01
Diabetes	14 (19.7)	13 (17.3)	0.71
Cerebrovascular diseases	10 (14.1)	4 (5.3)	0.07
Dementia	9 (12.7)	1 (1.3)	<0.01
Malignancies	8 (11.3)	10 (13.3)	0.70
Gastrectomy	6 (8.5)	3 (4.0)	0.26

NHTB—nursing- and healthcare-associated tuberculosis; CTB—community-acquired tuberculosis.

**Table 4:** Clinical courses of patients with NHTB and CTB.

Items	NHTB (n = 71)	CTB (n = 75)	*P*-Value
Period of hospitalization (days)	77.6 ± 42.5 ^a^	94.0 ± 42.6	0.02
Discontinuation of treatment (%)	25 (35.2)	17 (22.7)	0.94
In-hospital death	28 (39.4)	3 (4.0)	<0.05
Death due to tuberculosis	26	2	
Death due to other causes	2	1	

^a^ Mean ± standard deviation. NHTB—nursing- and healthcare-associated tuberculosis; CTB—community-acquired tuberculosis.

## Abbreviations

CRP	C-reactive protein
CTB	community-acquired tuberculosis
JRS	Japanese Respiratory Society
NHCAP	nursing- and healthcare-associated pneumonia
NHTB	nursing- and healthcare-associated tuberculosis
PS	performance status
TB	tuberculosis
WBC	white blood cell
